# Helping Patients Communicate With Oncologists When Cancer Treatment Resistance Occurs to Develop, Test, and Implement a Patient Communication Aid: Sequential Collaborative Mixed Methods Study

**DOI:** 10.2196/26414

**Published:** 2022-01-12

**Authors:** Anne Brédart, Aude Rault, Johanna Terrasson, Etienne Seigneur, Leanne De Koning, Elisabeth Hess, Alexia Savignoni, Paul Cottu, Jean-Yves Pierga, Sophie Piperno-Neumann, Manuel Rodrigues, Carole Bouleuc, Sylvie Dolbeault

**Affiliations:** 1 Psycho-Oncology Unit Institut Curie Paris Sciences et Lettres Research University Paris France; 2 Psychopathology and Health Process Paris University Boulogne Billancourt France; 3 Research Centre Paris Sciences et Lettres Research University Institut Curie Paris France; 4 Direction Recherche Ensemble Hospitalier, Data Management Unit Biometry Department Institut Curie Saint-Cloud France; 5 Medical Oncology Department Institut Curie Paris France; 6 Faculty of medicine Paris University Paris France; 7 Département Interdisciplinaire de Soins de Support pour le Patient en Oncologie Department of Supportive Care Institut Curie Paris France; 8 Research Centre in Epidemiology and Population Health (CESP) INSERM, U1018 University Paris-Sud Villejuif France

**Keywords:** cancer resistance, physician-patient communication, question prompt list, patient participation, collaborative research, mixed methods

## Abstract

**Background:**

Most cancer-related deaths result from disseminated diseases that develop resistance to anticancer treatments. Inappropriate communication in this challenging situation may result in unmet patient information and support needs. Patient communication aids such as question prompt lists (QPLs) may help.

**Objective:**

This study aims to develop and pilot-test a specific QPL in the following two contrasting clinical contexts in France after cancer resistance has developed: triple-negative and luminal B metastatic breast cancer (MBC) and metastatic uveal melanoma (MUM).

**Methods:**

A sequential study design with a mixed methods collaborative approach will be applied. The first step aims to build a specific QPL. Step 1a will explore oncologist-patient communication issues from oncology professionals’ interviews (n=20 approximately). Step 1b will appraise information and support needs experienced by patients with MBC or MUM both quantitatively (n=80) and qualitatively (n=40 approximately). These data will be used to develop and pilot-test a QPL specific to patients with cancer experiencing initial or acquired resistance to treatment. We expect to obtain a core QPL that comprises questions and concerns commonly expressed by patients with resistant cancer and is complemented by specific issues for either MBC or MUM cancer sites. In step 1c, 2 focus groups of patients with any type of metastatic cancer (n=4) and health care professionals (n=4) will be conducted to revise the content of a preliminary QPL and elaborate an acceptable and feasible clinical implementation. In step 1d, the content of the QPL version 1 and implementation guidance will be validated using a Delphi process. Step 2 will pilot-test the QPL version 1 in real practice with patients with MBC or MUM (n=80). Clinical utility will be assessed by comparing responses to questionnaires administered in step 1b (QPL-naive historical control group) and step 2 (QPL intervention group).

**Results:**

This study received grants in March and December 2019 and was approved by the French national ethics committee in July 2019. As of October 2021, interviews with oncology professionals have been conducted and analyzed (N=26 to reach saturation), and 39 and 27 patients with MBC and MUM, respectively, have been recruited.

**Conclusions:**

A clinically and culturally tailored QPL is expected to facilitate patients’ participation in consultations, improve oncologists’ responses to patients’ information and support needs, and thus foster patients’ psychological adjustment to the diagnosis and follow-up of cancer resistance to treatment.

**Trial Registration:**

ClinicalTrials.gov NCT04118062; http://clinicaltrials.gov/ct2/show/NCT04118062

**International Registered Report Identifier (IRRID):**

DERR1-10.2196/26414

## Introduction

### Background

Most advanced cancers eventually develop resistance to anticancer therapies, ultimately leading to the progression of the disease, symptom burden, and death [1]. Resistance may occur very early in the course of disease (often called *primary resistance*) or, in most instances, is acquired under long-term treatment exposure after a favorable initial response (secondary resistance).

Cancer progression, resulting from resistance to therapy, often indicates the onset of an advanced disease that will not be cured, although life expectancy may still be months or years with adequate treatment. This clinical situation represents a critical moment in the course of cancer care. Oncologist-patient communication is then particularly challenging. When resistance occurs, patient information on the severity of the disease, its prognosis, and the therapeutic options must be conducted with utmost subtlety. Patients are informed about alternative cancer treatments, expectations about their effectiveness, and side effects. This information is anxiety provoking, often eliciting the need for emotional support.

Information on prognosis and treatment outcomes is important for achieving a shared perception of the disease status and treatment goals between patients and oncologists [2-4]. A general population survey performed in 7 European countries indicates that 73% of citizens prefer to be informed in case of a poor disease prognosis (≤1 year to live) [5]. In the advanced cancer setting, most patients want a realistic understanding of their current condition and life expectancy; however, not all wish to receive exact or definitive time frames [2,6-9]. Oncologists have difficulty appraising patients’ information preferences [10]. Information on prognosis is often lacking [11], and patients do not understand that the treatment provided is not likely to cure their cancer [12].

In addition, patients with advanced cancer do not participate as much as they wish during consultations [8,13]. Possible explanatory factors include patients forgetting questions, doubting the legitimacy of asking, expressing concerns indirectly, fear of the possible pejorative answer, and a lack of physicians encouraging their questions [14-16]. Patients may also present different needs and expectations, which depend on the time in their disease course [17] or factors such as their attentional coping style or sociodemographic background [10,18,19]. Discordance between the patient’s and oncologist’s perception of treatment aims and the disease timescale may result in medical decisions that do not align with life goals that are important to patients [20,21]. This leads to patients’ greater psychological distress [22].

Patient-centered communication is the cornerstone of high care quality [23]. This allows physicians to better respond to patients’ information and support needs. To this end, guidelines to improve communication skills of health care professionals are available in many countries [24-28]. Effective physician-patient communication may decrease patients’ anxiety, sustain hope [29], and increase satisfaction with care [30-32]. Patient-focused communication aids have also been developed to complement physician communication skills training [18,30-32]. These interventions are designed to enhance patients’ participation in the consultation and thus may increase physicians’ awareness and timely accommodation of their needs and expectations [33-36].

### Question Prompt Lists

Among patient-focused communication aids, question prompt lists (QPLs) [37-43] may help patients express their information and support needs according to their wishes. A QPL includes a structured list of questions given to the patient before the consultation. This intervention drives patients to more frequently ask questions and express concerns, especially regarding disease prognosis, and may enhance patients’ recall of information and satisfaction with care [38,39,41,42,44-48].

Most QPLs that are already available address early cancers [39,49] or palliative care [41,50-52], specific clinical situations (eg, early breast cancer [53], esophageal cancer [54,55], and myelodysplastic syndrome [56]), care circumstances (eg, clinical trials [57,58]), and populations (eg, older patients with cancer [58] or cultures or ethnicity [53,59])

A QPL for oncology consultations taking place during the first treatment lines after cancer resistance has developed seems to be lacking. Such a QPL is expected to contain questions or concerns related to the following issues: disease severity, extent of spread, future course, medical tests, treatment options, course of symptoms and side effects [60], likelihood of cure, primary goal of cancer care, expected treatment effectiveness and life expectancy [61], psychological needs [62], self-management [63], and cancer care provision and organization [64]. A QPL designed for advanced cancer care in Australia and adapted in the United States [37] and in the Netherlands [18] will serve as a basis.

In Western countries, a shift has been witnessed in models of care from a paternalistic to a patient-centered approach, tailoring information to patient preferences and wishes [65]. However, for example, little information exists on French patients’ wishes to receive information on prognosis [56] and on the concordance between oncologists’ information provision and the expectations of patients with cancer. In a previous study with patients receiving palliative care, we observed that addressing disease prognosis was seen as particularly difficult for clinicians [66]. The cultural adaptation of a QPL may be necessary not only in terms of content but also in terms of implementation modalities. Theoretically, QPLs are simple and cost-effective; however, their acceptability and implementation feasibility should also be verified cross-culturally. Therefore, optimal modalities and procedures of applying this tool in real oncology practice in France will also be explored and delineated. It is possible that a patient coaching or education group intervention may be needed as a complement.

### Clinical Setting

The QPL will be developed in the following two clinical contexts, which are prone to cancer resistance, and contrasted in terms of epidemiology, life expectancy, long-term treatment options, and expected effectiveness: (1) triple-negative and luminal B metastatic breast cancer (MBC) and (2) metastatic uveal melanoma (MUM), both within the first 3 lines of anticancer treatment after treatment resistance has occurred.

Triple-negative and luminal B MBC represents approximately 15% and 50% of all breast cancer cases, respectively [67], and recurs with distant metastasis in approximately 30% of early-stage patients [68]. MBC is an incurable disease, with a median overall survival of 3 years and a 5-year survival rate of 25% [69]. Patients with MBC are generally followed by their oncologist for years, punctuated by occurrences of treatment resistance for which a new treatment regimen may be offered. This implies successive disclosures of the progressing disease and discussions of alternative treatment regimens [70].

Uveal melanoma is a rare cancer [71]. Up to 50% of patients with uveal melanoma develop metastases, mostly in the liver, within a median time of 2 to 3 years [72]. Once metastasis occurs, the median overall survival ranges from 9 to 12 months because of the lack of effective treatment options [73]. Most patients with MUM are referred to a medical oncologist after a multidisciplinary tumor board meeting. Medical oncologists then inform patients about metastatic progression and possible treatment alternatives. The dismal prognosis and rapid evolution of the disease are challenging aspects in the communication between oncologists and patients. A discrepancy in knowledge appears when the medical oncologist is aware of the patient’s poor prognosis while the patient is still in good shape and does not expect such quick, fatal outcomes.

### Objective

We propose an original design for the development of a complex intervention based on the Medical Research Council framework [74].

The specific aims are (1) to develop the content of a QPL for adults diagnosed with resistant cancer, who are still eligible for disease-targeted treatment, and (2) to pilot-test the implementation of this intervention in oncology consultations in a French cancer setting. During this process, the implementation will be prepared by investigating potential obstacles and facilitation strategies from the outset.

We expect to obtain a core QPL comprising questions and concerns that patients in the treatment resistance context might generally want to express at the oncology consultation. A common *core* QPL will be complemented with subsections containing specific issues for either the triple-negative and luminal B MBC or the MUM cancer sites.

## Methods

### Patient and Public Involvement

This psychosocial study is embedded in an overall medical research program performed within the Institut Curie Integrated Cancer Research Site—*Site de Recherche Intégrée sur le Cancer*. In France, this framework is equivalent to a comprehensive cancer center and has been labeled by the French National Cancer Institute. This research program deals with treatment resistance in triple-negative and luminal B MBC and MUM. A patient and partner representative committee was created with the objective of fostering public debate and integrating patients’ voices into research development, conduct, and dissemination. This committee was involved in the choice of the psychosocial study research question. Outcome measures were selected according to priorities, experience, and preferences. They found it particularly relevant and timely to address communication difficulties between the oncologist and the patient when treatment resistance occurs. Since 2017, their involvement has taken place at regular steering committees and brainstorming meetings. They provided feedback on this study protocol; the information and consent forms; and the content, format, and burden of the questionnaires.

### Study Design

**Figure 1:**
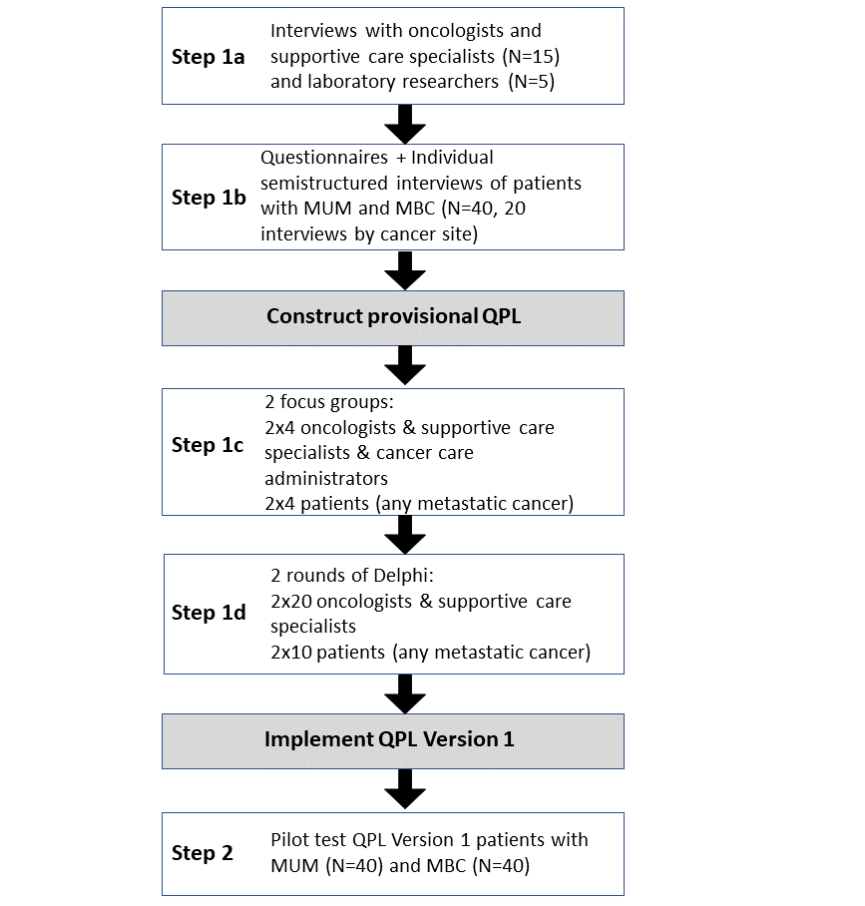
Sequential study design. MBC: metastatic breast cancer; MUM: metastatic uveal melanoma; QPL: question prompt list.

As shown in Figure 1 and Table 1, we will adopt a sequential design and mixed methods approach [75,76], evolving in successive steps and building on qualitative (individual and focus group interviews) and quantitative (standardized questionnaires, acceptability assessment, and Delphi process) data collection and analysis. It will involve the collaboration of adult patients with MBC or MUM, patients with any type of metastatic cancer (ie, *expert patient*, defined as “individuals with an experience of cancer diagnosis & treatment and educated knowledge of their disease and treatment trajectory” [77,78]), oncologists, supportive care specialists, cancer care administrators, and laboratory researchers dealing with cancer resistance [79].

**Table 1:** Sequential collaborative mixed methods approach.

Steps		Aim	Method	Sources or population
**Step 1: QPL^a^ development**		To develop a QPL for the treatment of resistant cancer in 2 contrasting clinical contexts: MBC^b^ (triple-negative and luminal B) and MUM^c^	Mixed methods	Professionals and patients
	Step 1a	To explore oncologist-patient communication issues (ie, difficulties, obstacles, and strategies) in the context of cancer resistance	Individual interviews	Professionals, that is, oncology physicians, supportive care specialists, and laboratory researchers (from bench to bedside)
	Step 1b	To explore communication difficulties and information and support needs that are experienced by patients with resistant cancer after an oncology consultation to initiate or follow a new disease-specific treatment after cancer resistance has developed	Standardized questionnaires Individual interviews	Patients with MBC (triple-negative and luminal B) or MUM within the first 3 lines after first cancer resistance^d^
	Step 1c	To revise the content of a QPL developed for patients with advanced cancer and elaborate an acceptable and feasible clinical implementation, potentially facilitated by a coaching intervention	Focus groups	Professionals and patients (any type of metastatic cancer)
	Step 1d	To validate the content of a QPL preliminary version adapted to cancer resistance and the MBC (triple-negative and luminal B) and MUM contexts (version 1) and the implementation guidelines	Delphi surveys	Professionals and patients (any type of metastatic cancer)
Step 2: QPL pilot testing		To pilot-test a QPL version 1: acceptability, feasibility, and potential clinical utility (effects)	Standardized questionnaires (same as in step 1b)	Patients with MBC (triple-negative and luminal B) or MUM within the first 3 lines after first cancer resistance

^a^QPL: question prompt list.

^b^MBC: metastatic breast cancer.

^c^MUM: metastatic uveal melanoma.

^d^Patients in step 1b comprise a historical control group to which the patients provided with the preliminary question prompt list in step 2 will be contrasted.

### Study Assessment and Procedures

The approval for the study was obtained from the French national ethics committee (ID-RCB: 2019-A01713-54). All patients will provide signed informed consent. The study, for which the acronym is HECTOR (Helping Patients Communicate With Oncologists When Cancer Treatment Resistance Occurs), is registered as NCT04118062 in ClinicalTrials.gov.

### Step 1: QPL Development and Implementation Guidelines

#### Overview

Step 1 focuses on determining the QPL content, structure, and format, as well as the clinical implementation modalities. This step will allow the definition of the clinical setting of cancer resistance and the factors that may facilitate or impede patients’ participation in oncology consultation. We aim to define a print brochure, along with a website and mobile app, offering a QPL perceived as attractive and satisfactory by patients and clinicians and that may be successfully used in the clinic (Table 1).

Interviews with patients and professionals will explore patient-oncologist communication issues in the context of cancer resistance. Moreover, study participants will be prompted to consider the relevance of a communication aid in the form of a QPL and complement this tool with patient coaching (ie, an intervention involving an interaction with a health professional or peer-support patient) and to prepare the operational implementation of this intervention in routine practice [80,81].

#### Step 1a: Oncologist-Patient Communication in the Clinical Management of Resistant Cancer

Individual semistructured interviews with oncologists, supportive care specialists, and laboratory researchers selected according to the inclusion and exclusion criteria listed in Textbox 1 started in July 2019.

**Professionals and patients’ eligibility criteria included at each step of the study.**

**Inclusion criteria**

- Professionals
- Oncology physicians (medical oncologist, oncology surgeon, radiation oncologist, and supportive care specialist), oncology laboratory researchers, and cancer care administrators
- Dealing with cancer resistance in solid malignancies
- Patients with metastatic breast cancer or metastatic uveal melanoma (MUM)
- Aged ≥18 years
- Diagnosed with resistant cancer
- Metastatic triple-negative or luminal B breast cancer or MUM
- Within the first 3 treatment lines after cancer resistance has developed
- Informed of cancer diagnostic and treatment resistance
- Patients with any type of metastatic cancer
- Aged ≥18 years
- With any type of cancer diagnosis

**Exclusion criteria**

- Professionals
- Physicians or laboratory researchers not involved in cancer resistance research
- Patients with metastatic breast cancer or MUM
- Unable to complete surveys in French
- Patients with any type of metastatic cancer
- Unable to participate in a group interview or complete a questionnaire survey because of physical, cognitive, or linguistic (French language) barriers

A purposeful sample is planned to allow the obtaining of a variety of perspectives on the clinical situation of cancer resistance [82]. Snowball sampling (ie, inclusion by approaching further participants through initial ones) will identify participants in different oncology hospitals or departments (France) from participants initially interviewed in 1 cancer center (Institut Curie, Paris).

Laboratory researchers will be solicited because of their outlook and up-to-date knowledge of cancer resistance research; they will be susceptible to anticipate new therapeutic regimens and their clinical translations.

An interview guide comprising open-ended questions will explore the following three central themes: (1) the definition of drug resistance and cancer resistance; (2) perceptions of oncologist-patient communication when the patient is diagnosed with resistant cancer, initial or acquired, after 1 or several treatment regimens; and (3) perceptions of oncologist-patient communication difficulties, obstacles, and facilitating strategies in the clinical context. The expected sample size based on data saturation is approximately 20 participants (Table 2) [83]. The duration of each interview is estimated to be between 30 and 45 minutes.

**Table 2:** Sample size by study population.

Population and data collection			Cancer resistance to treatment (before consultation)	
			Historical control (step 1; N=80)	QPL^a^ (step 2; N=80)
**Number of patients**				
	**MUM^b^** **within the first 3 treatment lines after cancer resistance has developed**			
		Questionnaires	40	40
		Individual interviews	20	—^c^
	**Triple-negative and luminal B MBC^d^** **within the first 3 treatment lines after cancer resistance has developed**			
		Questionnaires	40	40
		Individual interviews	20	—
**Number of professionals**				
	**Oncologists or supportive care specialists or cancer care administrators**			
		Individual interviews	15	—
		2 focus groups	8^e^	—
		2-round Delphi	40^f^	—
	**Laboratory researchers**			
		Individual interviews	5	—
**Number of patients with any type of cancer diagnosis**				
	**Any cancer**			
		2 focus groups	8^e^	—
		2-round Delphi	20^g^	—

^a^QPL: question prompt list.

^b^MUM: metastatic uveal melanoma.

^c^Not available (no individual interview in step 2).

^d^MBC: metastatic breast cancer.

^e^n=2×4.

^f^n=2×20.

^g^n=2×10.

#### Step 1b: Patients’ Communication Experience and Information and Support Needs in the Oncology Consultation Dealing With Treatment Resistance

##### Overview

A cross-sectional assessment of patients’ communication needs in the context of cancer resistance will then be performed. Patient enrollment started in January 2021 and will take place over 1 year.

Consecutive patients will be identified via lists of oncology consultation agendas in an oncology center (Institut Curie, Paris). If they respond to the inclusion and exclusion criteria listed in Textbox 1, they will be invited to participate in the study.

Patients’ sociodemographic data (age, gender, educational level, and marital, parental, and professional status) and clinical data (date of initial diagnosis, disease recurrence and metastatic occurrence, stage, previous and current disease-targeted treatments, and supportive care interventions) will be collected from patients or medical records to describe the study population.

##### Qualitative Assessment

Individual semistructured interviews will be proposed to a random subsample of these patients to specify in greater depth and detail the nature and temporality of communication experience, difficulties, and needs when confronted with cancer resistance.

On the basis of data saturation [83,84], an expected number of approximately 20 patients per cancer site will be interviewed no later than 1 month after they complete the questionnaires (Table 2).

The interview guide, comprising open-ended questions, will explore the following three central themes: (1) patients’ actual experience of communication with the oncologist; (2) their expectations, preferences, met and unmet information, and support needs (retrospectively and prospectively); and (3) their opinions about a specific QPL to help them communicate with oncologists. Individual interviews are estimated to last between 30 and 45 minutes.

##### Quantitative Assessment

Standardized questionnaires will be completed by patients to describe their unmet information and support needs while facing cancer resistance in the MBC and MUM settings. These questionnaires will also be used to obtain preliminary data, albeit limited to the potential clinical usefulness of the designed QPL. This will be performed by comparing responses from patients at step 1b (QPL-naive group) with clinically similar patients at step 2 (QPL intervention group).

As information needs may depend on patients’ characteristics [10,18], these outcomes will be assessed according to patients’ sociodemographic (ie, age, gender, educational level, and marital status) and psychological correlates (ie, beliefs, preference for information, level of distress, and coping strategies). To address these outcomes and correlates, patients will be invited to complete standardized questionnaires, as detailed in Table 3.

**Table 3:** Standardized questionnaires.

Measures of QPL^a^ potential clinical benefits			Factors assessed
**Outcomes**			
	1 item of the PTPQ^b^ [61]	Satisfaction with the quality of information received about prognosis and treatment	
	EORTC QLQ-INFO25^c^ information about the disease, medical tests, and treatments scales (items 31-43) and satisfaction with information items (52-55) [60]	Perception of information received about the disease, medical tests, and treatments Satisfaction with information	
	SCNS-SF34^d^, Psychological (items 6-14 and 17) and Care and Support needs (items 18-22) [85]	Perception of unmet psychological (eg, anxiety, fear of cancer spreading, and uncertainty) and care and support needs (eg, reassurance and sensitivity to feelings and emotional needs)	
**Correlates**			
	12 items of the PTPQ [61]	Beliefs regarding the likelihood of cure, the importance and helpfulness of knowing about prognosis, and the primary goal of cancer care Preference for information about treatment	
	HADS^e^ [86]	Anxiety and depression	
	Brief COPE^f^ [87]	Coping strategies	

^a^QPL: question prompt list.

^b^PTPQ: Prognosis and Treatment Perceptions Questionnaire.

^c^EORTC QLQ-INFO25: European Organization for Research and Treatment of Cancer Quality of Life Questionnaire–Information Module.

^d^SCNS-SF34: Supportive Care Needs Survey–Short Form.

^e^HADS: Hospital Anxiety and Depression Scale.

^f^COPE: Coping Orientation to Problems Experienced.

Patients’ perception of prognosis and treatment will be assessed using the Prognosis and Treatment Perception Questionnaire (PTPQ) [61]. The PTPQ assesses beliefs regarding (1) the likelihood of cure, (2) the importance and helpfulness of knowing about prognosis, (3) the primary goal of cancer care, (4) preference for information about treatment, and (5) satisfaction with the quality of information received about prognosis and treatment (Table 3). This questionnaire has been translated into French and pilot-tested according to the European Organization for Research and Treatment of Cancer Quality of Life Group guidelines [88].

The perception of information received about the disease, medical tests and treatments, and satisfaction with overall medical information will be measured by the European Organization for Research and Treatment of Cancer Quality of Life Questionnaire–Information Module (EORTC QLQ-INFO25) scale [60]. The perception of unmet psychological (eg, anxiety, fear of cancer spreading, and uncertainty) and care and support needs (eg, reassurance, sensitivity to patients’ feelings, and emotional needs) will be measured by the Supportive Care Needs Survey–Short Form (SCNS-SF34) [85].

Additional patient information will include coping strategies (usual strategies when facing stressful life events) and distress (during the past week), as measured by the Brief Coping Orientation to Problems Experienced [87] and the Hospital Anxiety and Depression Scale [86] questionnaires, respectively.

Questionnaires will be completed within 2 weeks of the consultation; if not completed within this time-lapse, 1 reminder will be made by telephone. Questionnaires not returned after 1 month will be considered missing.

As indicated in Table 2, a sample size of 80 patients (40 patients by tumor site) is planned. Sample sizes up to 40 per group are expected to provide estimates that are precise enough to assess the feasibility of QPL use and obtain preliminary clinical data for further randomized controlled trials of this tool [89].

#### Step 1c: Content and Implementation of the QPL in Routine Practice

From steps 1a and 1b, a core QPL comprising issues (ie, questions and concerns) commonly expressed by patients with resistant cancer, complemented by specific issues for either MBC or MUM cancer sites, will be developed. Issues to compose this QPL will be selected based on descriptive analyses of patients’ responses to relevant items of the PTPQ, EORTC QLQ-INFO25, and SNCS-SF34 questionnaires. For example, if ≥50% of patients report that they received little or no information about the spread of their illness from the EORTC QLQ-INFO25 and are dissatisfied with the information provided, this issue will be prioritized while composing the QPL. Other issues will be similarly selected based on responses to items of the SCNS-SF34 (50% of patients reporting medium or high unmet needs) or the PTPQ (50% reporting that the quality of information on treatment options received from the oncologist was fair or poor). These quantitative data will be considered in conjunction with the qualitative interview data. A thematic content analysis will help identify issues that patients would like to address more frequently during oncology consultations dealing with cancer resistance.

Focus groups will be implemented to discuss the provisional QPL. On the basis of research on sample size calculation for the content analysis of qualitative interviews [83], we aim to conduct 2 focus groups of approximately 8 different participants each (patients with any type of metastatic cancer, oncologists, supportive care specialists, and cancer care administrators from various oncology centers or departments in France), approached through snowball purposive sampling. Participants will be identified from contacts of *expert* patients (patient university, Institut Curie *Site de Recherche Intégrée sur le Cancer* patient and partner representatives, and cancer patient associations) and oncology professionals (UNICANCER oncology professionals’ network and French Association for Supportive Care—Association Francophone pour Soins Oncologiques de Support).

The group interview guide will address the following two central themes: (1) the appropriateness (adequacy, relevance, and importance for treatment resistance in oncology generally and in triple-negative and luminal B MBC and MUM specifically) and acceptability (satisfaction, anxiety-provoking, intrusiveness, irrelevance, and incompleteness) of the QPL content (questions, concerns or emotions, and narratives or testimonies), structure (logical order, length, and complexity), and format (paper, website, and app) and (2) the feasibility (obstacles and facilitating strategies such as complementary coaching, formal implementation blueprint, educational materials, and audit and feedback [90]) and optimal circumstances and procedures (when: timing of provision or access; where: hospital or home; how: text or video; who: coach or health educator expertise) for implementing the QPL in real-world clinical practice and ensuring its adoption and sustainability. The internationally designed QPL for patients with advanced cancer has been revised and further developed for the cancer resistance context by keeping, removing, or adding items according to their relevance and importance generally for treatment resistance in oncology and specifically for triple-negative and luminal B MBC and MUM.

Each focus group will be conducted via Microsoft Teams videoconference to facilitate participation and will last an estimated 90 to 120 minutes.

#### Step 1d: Content Validation With the Delphi Process

To facilitate the collection of individuals’ feedback on the provisional QPL version, a 2-round web-based Delphi process [91] will be performed involving participants through snowball purposive sampling. Participants in the focus groups will be offered the opportunity to participate in the consensus method. Other eligible participants (see criteria in Textbox 1) will be solicited according to the same recruitment methodology as for the focus groups.

The Delphi survey assesses (1) each QPL item (instructions, questions, concerns or emotions, and narratives or testimonies) in terms of content appropriateness, formulation clarity, structure, format, and acceptability on a 5-point Likert agreement scale and (2) the feasibility of a complementary coaching intervention and implementation guidance.

The procedure comprises successive evaluations according to the following steps:

- Participants will indicate their level of agreement on the relevance and clarity of each proposed item of the QPL (ie, instruction sentences and questions or concerns) and implementation guidance (ie, ideas or sentences) using a 5-point Likert scale (ranging from 1=*strongly disagree* to 5=*strongly agree*). The overall length and clarity of the tool and guidance will also be assessed.
- Participants will comment and propose changes or additions to the QPL and guidance if required based on the following questions addressed to patients (professionals):

What would you (your patients) have liked to ask?

What questions do you (your patients) often not ask, that you (I) wish you (they) would ask?

- The research team will analyze the data obtained. It will then identify the issues on which there is consensus and make possible modifications and additions based on the participants’ comments.
- Following the first evaluation, the modified items will be submitted to a second evaluation by participants who will be invited to rate each question, concern, or sentence on a 5-point Likert scale, and responses will be rated as *essential* or *important*; validation will be determined by an a priori threshold of ≥4.0.
- Following the second evaluation, QPL version 1 and guidance will be validated in its final version.

A total of 2 rounds of Delphi surveys administered via REDCap (Research Electronic Data Capture; Vanderbilt University) software will be performed, including 30 participants responding to the first survey on a first QPL version and then the second survey on a QPL version revised from the initial survey responses and comments.

### Step 2: QPL and Implementation Pilot Testing

The second step of the study pilot tests the QPL version 1 for its acceptability, feasibility, potential clinical utility, and sustainability. Consecutive patients (n=80) responding to the same eligibility criteria as in step 1b will be recruited. They will receive the QPL version 1 to prepare their subsequent oncology consultations, either a consultation during which the diagnosis of cancer resistance is communicated or in the course of a new disease-targeted treatment follow-up consultation. They will be invited to complete the same standardized questionnaires as in step 1b.

Additional questions will address the QPL acceptability in terms of uptake (the QPL has been read before the consultation), use (QPL items have been raised during the consultation), and patients’ and clinicians’ perceived helpfulness and satisfaction with this tool and its use in clinical practice.

### Data Analysis

#### Qualitative Data

All interviews will be audiotaped, transcribed, and identified using an alphanumeric log. A thematic analysis will be conducted using the RQDA (R package for the qualitative data analysis) software (version 2.15.2; 2012-10-26). A total of 2 junior (JT and AR) postdoctoral health psychologists and 1 senior (AB) postdoctoral health psychologist will code the transcripts. The analysis of thematic content will allow both the frequencies of responses for each category to appear and the meaning of the responses associated with each category to emerge [92,93]. A coding grid will be constructed from 2 complementary processes; (1) a pre-established code based on the research objectives and the semidirected interview guide will be elaborated to create broad coding categories and subcategories, and (2) a third of the interviews will be coded, using an emergent coding method, to test and modify the grid. Following an iterative process, several rounds of analysis will be conducted to stabilize the coding sheet. Finally, double interjudge and intrajudge coding will be conducted to ensure the reliability and independence of coding.

A similar content analysis will be performed for focus group interviews, with coding based on themes related to the content, format, and clinical implementation guidance of the QPL.

#### Quantitative Data

Statistical analyses will be conducted using SPSS software (version 27.0; IBM Corp). Standardized questionnaires used in steps 1b and 2, and the Delphi surveys, will be analyzed descriptively in terms of missing data, response frequency, mean and SD, median, and range.

In step 1b, responses to items of the PTPQ, EORTC QLQ-INFO25, and SCNS-SF34 will determine the prevalence of communication needs in the cancer resistance setting and thus help prioritize communication issues to compose the QPL.

The step 1b group (QPL naive) will comprise a historical control of patients from step 2 (QPL intervention group). Quantitative data collected from standardized questionnaires at step 1b will be compared with the data collected at step 2. Patients will be consecutively included in steps 1b and 2; therefore, they are expected to be clinically similar; however, sociodemographic and clinical data between these groups will be compared to check their similarity. Bivariate analyses of outcome measures will be performed to preliminarily assess the potential clinical usefulness of the QPL. Bivariate analyses will also be performed to explore patients’ satisfaction with information and support needs in relation to their sociodemographic and psychological characteristics (distress and coping strategies) in step 2 samples, overall and by cancer site.

## Results

This study received grants from the Ile-de-France CancerôPole (2019-1-EMERG-14-ICH-1; March 2019) and from the *Fondation de France* (2019 number 00101610; December 2019) and was approved by the French national ethics committee in July 2019. As of December 2020, to reach data saturation, 26 oncology professionals’ interviews have been conducted and analyzed. As of October 31, 2021, a total of 40 and 31 patients with MBC and MUM have been recruited, 20 and 20 have been interviewed, and 39 and 28 have completed questionnaires, respectively.

## Discussion

### Principal Findings

This protocol describes a study using an innovative, sequential, mixed methods approach and involving patients as well as oncology professionals to collaboratively develop a QPL for cancer resistance in the French cultural context.

This study will be undertaken in 2 clinical settings prone to cancer resistance and contrasted in terms of epidemiology, life expectancy, long-term treatment options, and expected effectiveness. The resulting QPL is expected to comprise core issues related to the cancer resistance context to which specific issues will be added if needed, according to the tumor site. The core QPL is expected to be applicable in other advanced cancer contexts.

QPLs seem effective in raising patients’ asking questions [38,42,94]; however, a complementary coaching intervention may be needed to further patients’ support [95,96,97]. Coaching is the provision of nondirective support by an individual (either in-person or remotely eg, by telephone or the internet) [98]. It is expected to help patients assess their information needs (ie, about treatment options such as disease-targeted treatment [standard or experimental], best supportive care, watchful waiting, and their benefits and harms) [18], prepare and rehearse questions [33], express their concerns or emotions [99], and prioritize issues to discuss during the consultation.

This study’s sequential collaborative mixed methods approach is innovative; the following methodological aspects are expected to be fruitful. First, a triangulation of perspectives is foreseen as it involves patients, clinicians, and researchers, and thus a better grasp of the specific realities of oncology consultation communication in resistant cancer care.

Second, the quantitative and qualitative data collection approaches are complementary, and the results will be sequentially and iteratively integrated. In-depth interviews with different protagonists who are experts in cancer resistance at their own level are expected to increase the appropriateness and acceptability of the tool. Considering the modalities and procedures of tool implementation from the outset is also meant to promote its adoption in routine practice. Focus group discussions and exploratory quantitative analyses will help decide for whom, when, and how the QPL will be implemented in clinical routine.

Third, the quantitative assessment allows the assessment of the specific information and support needs that are experienced when faced with cancer resistance. These data may be compared between 2 groups: before (step 1b) versus after (step 2) the availability of the QPL. This will offer initial information on the clinical utility of the tool tailored to cancer resistance in the French cultural context.

Owing to consecutive sampling, information will be available about the number of patients willing to use the QPL and the number of oncologists who will engage in its use during consultations [92]. Furthermore, the resulting QPL will be pilot-tested on outcomes such as patients’ beliefs about primary cancer treatment goals or satisfaction with the information provided about prognosis and treatment, which are important [93] and previously lacking effect measurement of QPL use [2].

Finally, focus group discussions will specifically elicit collaborative work. The reactions and proposals of various appropriate persons [100] will help prepare the modalities of QPL use in routine practice and promote its long-term adoption [101], that is, the tool use in real-world clinical practice [102]. Moreover, the Delphi process aims to reach consensus among patients and oncology professionals on a QPL version 1 and explicit guidance for clinical implementation [103,104]. We anticipate that these persons will have diverging views and opinions about the tool [91]; thus, a consensus will have to be developed.

This protocol has several limitations. The methodology described herein is an innovative but long process. It may not be systematically applied as a new clinical context in need of a QPL. However, this study offers the opportunity to reveal unnecessary steps and thus indicate an optimized process for future research.

We focus on the communication between oncologists and patients; however, other oncology professionals such as nurses or psychologists may also play an important role in responding to patients’ information and support needs; therefore, further research is needed to address their specific role in addressing these needs. In addition, patients’ relatives may also present their specific information and support needs and may interact with the patient when facing this information. The QPL may also be useful to them, as such or adapted, and this must be evaluated.

The assessment of patients at step 1b and step 2 of this study is cross-sectional; therefore, we also need to consider that information is not collected on the same individual patients over their evolving needs and information processing.

### Dissemination

A clinically and culturally specific QPL complemented, if necessary, by a coaching intervention is expected to facilitate patients’ participation in oncology consultations to improve oncologists’ responses to their information and support needs. This tool allows patients to control the provided information according to their wishes and thus respects their potential ambivalence and need for hope. Digital health interventions provide patients with evidence-based interventions through software apps. The availability of QPLs through these technologies is increasing [55,105,106]. Digital health interventions are easily accessible to patients from their homes through mobile devices or websites. Studies suggest that they are cost-effective, increase uptake by patients and clinicians, and provide clinical benefits [107]. Therefore, this French-adapted QPL will be available not only in paper form (brochure) but also as an app (MyCurie app) and on the institutional website. It will contain information about the purpose, interests, and modalities of use of the brochure or web document. Moreover, studies have shown the importance of clinicians’ endorsement of the tool, encouraging its use by patients; therefore, particular attention will be given to an implementation guide that recommends communication about this tool during the consultation. Patient and partner representatives of the Institut Curie will be invited to attend the local public and scientific conferences planned to communicate about this study. They will be solicited for their feedback on articles to disseminate results nationally and internationally through popular or scientific journals.

### Conclusions

This research proposes an original methodology to adapt and further develop a QPL for patients with resistant cancer and enable its implementation in the French cultural context. It is expected to facilitate patients’ expression of questions, concerns, and emotions and, in that way, improve oncologists’ responses to their information and support needs. Clinically, this study will also improve the understanding of patients’ and clinicians’ experiences, difficulties, obstacles, and strategies in discussing prognosis and treatment options in the first anticancer treatment lines after cancer resistance has developed. Methodologically, it will be possible to infer an efficient method for designing and guiding the implementation of communication aids for patients with advanced cancer.

## Abbreviations

EORTC QLQ-INFO25: European Organization for Research and Treatment of Cancer Quality of Life Questionnaire–Information Module
HECTOR: Helping Patients Communicate With Oncologists When Cancer Treatment Resistance Occurs
MBC: metastatic breast cancer
MUM: metastatic uveal melanoma
PTPQ: Prognosis and Treatment Perception Questionnaire
QPL: question prompt list
REDCap: Research Electronic Data Capture; Vanderbilt University
SCNS-SF34: Supportive Care Needs Survey–Short Form
